# Effects of nine weeks isokinetic training on power, golf kinematics, and driver performance in pre-elite golfers

**DOI:** 10.1186/s13102-017-0086-9

**Published:** 2017-12-11

**Authors:** James Parker, Charlie Lagerhem, John Hellström, M. Charlotte Olsson

**Affiliations:** 1 The Rydberg Laboratory for Applied Sciences, School of Business, Engineering and Science, Halmstad University, Box 823, 301 18 Halmstad, Sweden; 2 Scandinavian College of Sport, Box 11365, 494 28 Gothenburg, Sweden; 3 Swedish Golf Federation, Stockholm, Sweden

**Keywords:** Golf biomechanics, Isokinetic training, Power, Driver performance, Kinematics, Performance gains

## Abstract

**Background:**

It has previously been shown that isotonic strength training can improve driver performance among golfers, though few studies have investigated effects of strength training on swing kinematics together with driver performance. In this study we investigated whether isokinetic rotational training could improve driver performance and swing kinematic variables amongst elite golfers.

**Methods:**

Twenty competitive pre-elite golfers (handicap better than −3.0), 13 men and 7 women, were split into two groups, one group received the isokinetic power training (IK) alongside their normal isotonic pre-season strength-training and the other group continued with their normal isotonic pre-season strength-training regime (IT). The IK group completed 12 sessions of isokinetic power training on a standing rotation exercise (10% body weight at 1 m/s) and barbell squat (25 kg plus 10% body weight at 0.5 m/s). The IT group continued with their normal isotonic pre-season strength-training regime. Participants were tested for rotational power, lower body power, golf swing kinematics, and driver performance before and after a nine-week training period.

**Results:**

After the nine-week training period both the IK and the IT groups increased their dominant side rotational force and power (effect sizes between 0.50–0.96) and magnitude based inference indicated that IK had a likely (> 80%) more beneficial increase in dominant side rotational force and power. For swing kinematics, IK had a likely (> 80%) more beneficial improvement in lead arm speed and acceleration compared to the IT group. For driver performance, IK had a possible (65%) beneficial effect on ball speed and likely (78%) beneficial effect on carry distance when compared to IT, whereas neither of the groups improved club head speed.

**Conclusion:**

In the present study on pre-elite golfers we found that 9 weeks of isokinetic training increased seated rotational force and power, peak arm speed and arm acceleration, ball speed, and carry distance more compared to isotonic training. Even though isokinetic training did not increase CHS, it did result in greater carry distance.

## Background

In competitive golf, the player’s ability to hit the ball a long distance affects the score in a positive way [1], and research highlights the importance of driving distance in relation to golf performance [2]. Initial ball velocity is dependent on centeredness of impact, club head velocity (i.e. magnitude and direction) and club face orientation [3–5]. Most research investigating driving performance in golf report a strong correlation between club head speed (CHS), initial ball velocity and thus carry (striking distance from impact to landing, excluding roll) [6, 7]. Recent research [8] reports kinematics, segmental sequence of action, and power output as other important factors impacting on driving performance. Thus, many golfers incorporate strength and power training into their training schedule in order to positively influence their swing kinematics. However, there is a paucity of research into these training strategies and a better understanding of how muscular strength and power training influence golf swing kinematics and driving performance in elite golfers is required.

Many studies have investigated the correlation between physical capacities and different measures of driver performance including CHS, ball speed, and driving distance [9–15]. These correlational studies only measure associations between variables and give little information about which variables can be improved through training. Longitudinal studies following changes in both physiological characteristics and driver performance in golf are better able to describe likely cause and effect relationships. Previous strength training interventions in different golf populations have included general strength exercises performing two to three sets of 10–12 repetitions performed two to three times a week over eight to 10 weeks and these studies found improvements in CHS and driving distance among recreational golfer [16–20], and in CHS amongst elite golfers [21]. There is a scarcity of research using fewer exercises and specific rotational exercises, rather than a multitude of general strength training exercises, despite the rotational nature of the golf swing. Only a few studies have included rotational tests or incorporated training exercises aimed to mimic the ballistic movements in the golf swing and they found marginal changes in CHS (1.5–1.6%) and in driving distance (4.3%) [16, 21]. Intervention studies using strength training in golf have received some criticism for a lack of research on skilled golfers and inclusion of control groups [1, 8]. The most common performance variable measured is CHS, however a review [8] found that ball speed was the driver performance variable most likely to increase from a strength training intervention. Outcome measures are mostly driver performance variables and do not include swing kinematic variable. Including measurement for swing kinematics would allow for a better understanding of the causality of improvement in driver performance from strength training.

Most strength and power intervention studies in golf have used driver performance variables as their outcome measurements whereas swing kinematic variables are rare [17]. The ability to generate and coordinate force through hips, torso, and shoulders during the downswing influences both swing kinematics and driver performance. It is therefore useful to understand how training influences not only driver performance but also the swing kinematics. Previous strength training [17], and motor control [22, 23] intervention studies which have investigated both changes in swing kinematics and driver performance have studied mainly male recreational level golfers (one female participant in total [23]). These studies found a significant reduction (−13%) in pelvis torso axial rotation at top of backswing (x-factor) [17]; a 2.7°- increase in pelvis-thorax separation during the early downswing (x-factor stretch) [22]; a 14% increase in pelvis, torso, and wrist velocity [17, 22]; and an increase in driver performance variables including CHS [17], ball speed [17], and carry distance [17, 22]. These studies indicate that for amateur golfers, reducing x-factor and increasing x-factor stretch have a positive impact on driver performance, however, if this holds true for high-level golfers as well is not known. Bulbulian et al. [23] included one woman out of seven participants and no other training intervention investigating swing kinematics has included women golfers. Cross-sectional studies comparing men and women golfers have found a number of differences in both physiological and golf swing kinematic variables. Horan et al. [24] studied movement variability and found that women exhibited higher variability in thorax-pelvis coupling mechanics during the downswing variability when compared to men but both groups showed similar end-point trajectory variability (hands and clubhead). Egret et al. [25] assessed differences in swing kinematics between experienced (average handicap −6.3) male and female golfers and found specifically hip and shoulder joint rotation angles at top of backswing differed between groups. Interestingly, they did not find any difference in clubhead speed. Zheng et al. [26], compared swing kinematics between male and female golfers on the PGA and LPGA tour and found differences in particular in maximum velocity of the wrists, right elbow extension, timing of left wrist extension velocity, and club head velocity. A greater X-factor stretch is assumed to make use of the stretch-shortening cycle (SSC), where a greater stretch of the torso musculature is assumed to allow for greater forces to be developed [1, 22]. Bulbulian et al. [23] reported a reduced electromyography activity in the torso and no change in driver performance after an intervention to shorten the backswing. These authors proposed that performance may have been maintained by increased loading of the shoulder musculature instead of torso musculature. Aside from the shoulder, performance maintenance may have been maintained by an increase in work done both below (lower body musculature) and/or above (upper body, shoulders, and arms) the torso. There is a paucity of research in physical training interventions on high-level golfers, which also study how the training intervention influence swing kinematics including the shoulder in addition to the pelvis-thorax segments.

Until recently, most strength and power intervention studies investigating isokinetic training in general, have used single joint movements [27] with only a few studies investigating multi-joint isokinetic training [28, 29]. In comparison to traditional isotonic training, isokinetic training, which is often performed at very low speeds (0.1–0.4 m/s), has the advantage that near maximal force can be exerted throughout the entire range of motion [28]. Similar to results from single joint isokinetic training, studies using multi-joint isokinetic training showed improved peak force and performance in dynamic movements [28, 29]. A recent study compared isotonic and isokinetic multi-joint (squat) training in different team sports athletes [28] and found that isokinetic multi-joint training improved select performance variables such as sprint and drop jump to a greater extent than traditional isotonic training. The use of multi-joint isokinetic training in a golf-specific movement and its influence on swing kinematics and driver performance among high-level golfers has not been studied previously. Thus, the purpose of this study was to investigate if isokinetic rotational and lower body strength training over 9 weeks is more effective than isotonic strength training in improving rotational and lower body power, pelvis-thorax and shoulder kinematics, and driver performance among high-level golfers.

## Methods

### Participants

Twenty intercollegiate golfers (13 men and 7 women) all competing at a national level or higher participated in the study. All subjects reported a handicap of −3.0 or better registered with the Swedish golf association at the time of the study. All subjects were free of musculoskeletal injuries for the previous 12 months and had a minimum of 3 years golf-specific strength training experience. The subjects did not all have the same swing coach. The study design was an open trial study since the participants could choose which group to belong to. Their choices were mainly based on individual travelling schedules and distance to the training facility during the investigation period.

There were no drop-outs in the study. This study was approved by the regional Swedish ethics committee (Lund, Dnr 2016/12) and all the participants gave written consent to participate in the study.

### Procedures

A training study was designed to investigate the difference between isotonic and isokinetic power training in golf. The load for the isokinetic group was controlled by a computerized robotic engine system (1080 Quantum Synchro, 1080 Motion AB, Lidingö, Sweden). The advantage of computerized robotic engine system is that isokinetic resistance can be applied to functional multi-joint exercises, such as golf specific rotational exercise and loaded squats [28].

### Training

The participants were divided into two groups, one group (*n* = 10, 6 men and 4 women) received the isokinetic (constant-speed) power training (IK) and the other group (*n* = 10, 7 men and 3 women) continued with their normal isotonic (constant load) pre-season strength-training regime and served as the reference isotonic group (IT).

The training period lasted 9 weeks, with 1 week of cessation in the middle of the period to accommodate competition calendars. Both groups resistance trained on average three times a week and had individualised programs of isotonic and isometric exercises performed both with free weights and body weight resistance as well as ballistic rotation exercises performed during the nine-week period. For the IK group, two isokinetic exercises replaced the ballistic rotation exercises and isotonic power exercises in their regular training program, where isokinetic power training was performed on average twice a week. The two isokinetic exercises, performed in a computerised robotic engine system, consisted of an isokinetic standing rotation exercise designed to replicate the golf swing and a loaded isokinetic squat. Both isokinetic exercises consisted of three sets of five repetitions where the isokinetic rotation exercise was performed with 10% body weight resistance and the speed set at 1 m/s concentrically and 4 m/s eccentrically. The loaded squat was performed with 25 kg barbell plus 10% body weight resistance and the speed set at 0.5 m/s concentrically and 4 m/s eccentrically.

#### Tests

*All tests were performed just before the beginning of the 9 week training intervention and within 1 week of the last training session.*

##### Power testing

Lower and upper body power tests were assessed using countermovement jumps with arm swing (CMJ), loaded squat jumps, and sitting abdominal rotation. Participants performed three repetitions on each test with 5 min rest between the repetitions; the repetition with the highest value on each weight was recorded.

The countermovement jumps were performed indoors, measurements of jump height were recorded with the use of infrared sensors (Ivar jump and speed analyzer, LN sport consult, Sweden). The subjects were instructed to stand in an upright position with their feet in a shoulder-width stance. The jump was initiated with a countermovement motion and continued in an explosive upward motion with the assistance of the arms. During the landing, the subjects aimed at finishing at the same position as the jump was initiated from.

Loaded squats jumps were performed with 20, 40 and 60 kg load on the shoulders. The subjects performed squat jumps from a 90° knee angle to full extension. Measurements of peak power were collected with the use of a linear encoder (MuscleLab, Model 4000, Ergotest Technology, Norway). For data analysis, only the 20 kg loaded squat jump was used since some participants were unable to conduct a technically correct and safe loaded squat jump at the higher loads.

Measurements of sitting isotonic abdominal rotational power were obtained in 1080 Quantum. The test used in this study consisted of a modified version of the test by Andre et al. [30]. The subjects were instructed to sit on a bench (height 46 cm, length 100 cm) with their feet on the floor. The bench was placed 125 cm from the handle which was set at shoulder height. The subjects were instructed to grip the handle and rotate their torso forcefully, with straight arms, and then slowly return to the starting position. Three repetitions on the left side and three repetitions on the right side were performed using a load of 10% of body weight. Rotating to the left was classified as the dominant side for a right-handed golfer as this is the same direction as they perform in the golf swing, and thus of main interest in this study. The highest peak force, power, and velocity value of the three repetitions on each side was used for later analyses.

Golf Swing analysis: All golf tests were performed at a driving range where subjects hit out onto a driving range. Swing kinematic data was using a four sensor electromagnetic motion capture system at 240 Hz (Polhemus Inc. Colchester, VT, USA) together with Advanced Motion Measurement software (AMM 3D, Phoenix, Arizona, USA) equipment previously used in golf research [31, 32]. The orientation of the right-handed orthogonal global coordinate system was such that the positive x-axis pointed parallel to the shot direction, the positive z-axis vertically upwards, and the positive y-axis forward from the right-handed golfer. The kinematics variables are described in Table 1 and placement of the sensors and digitization are described in Table 2. Thorax and pelvis rotations were calculated using the joint coordinate system method [33]. The lead arm segment was calculated using the humerus joint coordinate system (first option) [34] relative to the thorax.

**Table 1:** A description of how swing kinematic variables were determined

	Definitions
Transition	Is determined as the point of lowest angular velocity for a segment, between initiation of the backswing and impact.
X-factor	The change in amplitude of spinal rotation (difference between thorax and pelvis rotation) at pelvis transition
X-factor stretch	The maximum increase in X-factor during the downswing
X-factor stretch rate	The average speed of X-factor stretch
Shoulder stretch	The change in amplitude of lead arm horizontal adduction between thorax transition and lead arm transition
Shoulder stretch rate	The average speed of shoulder stretch
Pelvis acceleration	The average acceleration of the pelvis between pelvis transition and pelvis peak speed
Thorax acceleration	The average acceleration of the thorax between thorax transition and peak thorax speed
Lead arm acceleration	The average acceleration of the lead arm between lead arm transition and lead arm peak speed, measured around the local Z-axis at the shoulder joint

**Table 2:** Placement of magnetic sensors and description of landmarks used to create each segment

Segment	Sensor placement	Landmarks used for segment digitization
Club	Below Ggrip	Top of grip.
		Hozel.
		Club head, bottom groove at heel.
		Club head, bottom grove at toe.
		Club head, top groove at toe.
Left arm	Posterior upper arm	Left acromion process.
		Lateral epicondyle
		Medial epicondyle.
Thorax/Upper-body	On T5	Left acromion process.
		Right acromion process.
		Right side mid thorax, high.
		Right side mid thorax, low.
Pelvis	Sacrum	Left greater trochanter.
		Right greater trochanter.
		The point above left greater trochanter.

The subjects used their own golf club and premium Callaway range balls and were told to aim at a target set approximately 350 m away from the striking zone. All subjects performed a golf specific warm up of their choice for a maximum of 10 min. Subjects were then instructed to hit 5 balls with their driver and use the swing that was as ‘normal ‘as possible, for example when playing from a tee on a standard par-4 hole. Between each shot, subjects were instructed to walk out of the tee (strike) area and wait for 30 seconds before commencing their pre-shot routine for the subsequent trial. We chose to use a five trial procedure primarily due to the participants’ time constraints, and such five trial procedures have been used in previous research [24, 35]. The swing where highest CHS was achieved was then used for subsequent analysis. Golf ball launch analysis: CHS, ball speed, and carry distance data were collected using a launch monitor (Trackman3e, v.3.2, Trackman, Denmark) placed 2.5 m behind the golf ball.

### Statistical analysis

**Fig. 1:**
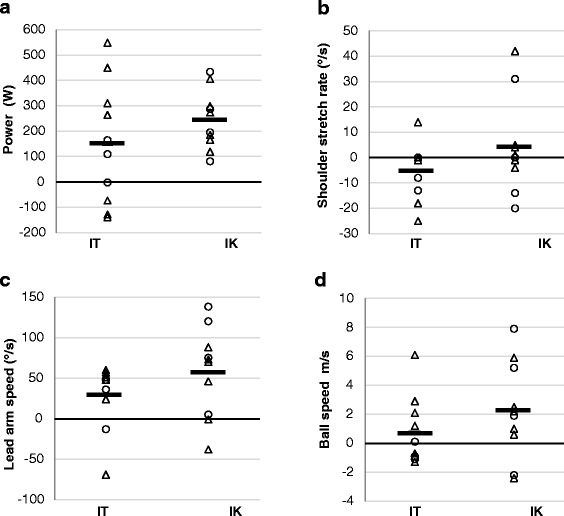
**a-d** Change after the nine-week training period in (**a**) seated rotational power, (**b**) shoulder stretch rate, (**c**) lead arm peak speed, and (**d**) ball speed after the nine-week training period for the isokinetic (IK) and isotonic (IT) groups. (Horizontal bars denote group means, circles signify women, triangles signify men)

All results are reported as mean ± standard deviation (SD). A probability level of 0.05 was used in this study. An independent t-test was performed to check for pre-test differences between IT and IK groups and between men and women. Since we could only recruit 7 female participants to this study, statistical analyses divided by sex, in the groups (IK = 4 and IT = 3 women) were not feasible. However, in the result figures below we show the individual values for each participant along with the group mean to visualise change for the men and women in the study. Magnitude based inference (MBI) was calculated using an online published spreadsheet [36], inferences were based on the disposition of the confidence limit for the mean difference to the smallest worthwhile change (0.2 between-subject SD). The probability that a change in testing score was beneficial, harmful or trivial was identified according to the magnitude-based inferences approach [37]. Descriptors were assigned using the following scales: 0–4.9% very unlikely; 5–24.9% unlikely; 25–74.9% possibly; 75–94.9% likely; 95–99.49% very likely; >99.5% most likely [38]. Within group standardized mean difference effect size (ES_w_) was calculated by using the mean change of the group (∆ IT or ∆ IK) in the numerator of the equation and using the pre-test pooled standard deviation in the denominator. Pre-test pooled standard deviation was calculated using pre-test values from the sample as whole (both IK and IT) [39]. Between-group standardized mean difference effect size (ES_b_) was calculated by using the difference between IK ES_w_ and IT ES_w_. An effect size of 0.20–0.50 are considered “small” in magnitude, those > 0.50–0.80 are “medium” and those above 0.80 are “large” as suggested by Cohen [40]. As well as presenting ES and results from MBI analysis this study presents standard deviations and figures describing change for each participant (Fig. 1a–d) to improve the transparency of the results.

## Results

Based on previous studies [24–26] showing cross-sectional differences in swing kinematics between men and women, we analysed the pre-training measurements variable for differences between sexes (Table 3). No differences were found before the training period began between men (*n* = 13) and women (*n* = 7) in swing kinematic-related variables (*p* > 0.05), whereas both power tests and performance measures differed between sexes (Table 3) as would be expected in highly skilled golfers [15, 21, 24, 26].

**Table 3:** Descriptive statistics and an independent t-test on physical, kinematics, and driver performance variables for men and women at the start of study

	Men (*n* = 13)	Women (*n* = 7)	Sig *p*-value
*Anthropometrics*			
Age (years*)*	21.8 ± 2.1	22.8 ± 1.8	0.30
Height (cm)	178.7 ± 7.3	169.7 ± 5.6	0.01
Weight (kg)	76.8 ± 11.0	65.7 ± 9.6	0.04
Hhandicap	+0.2 ± 1.5	+0.7 ± 1.0	0.40
*Tests for power*			
CMJ (cm)	43.7 ± 7.1	35.0 ± 5.5	0.01
Seated rotation dominant side			
Power (w)	793.8 ± 246.9	352.6 ± 96.8	0.00
Velocity (m/s)	3.5 ± 0.6	2.6 ± 0.3	0.00
Force (N)	261.8 ± 4.4	163.0 ± 27.2	0.00
Seated rotation non-dominant side			
Power (w)	812.3 ± 231.7	366.9 ± 115.3	0.00
Force (N)	258.4 ± 36.6	169.3 ± 31.3	0.00
Velocity (m/s)	3.3 ± 0.6	2.6 ± 0.4	0.01
*Kinematics*			
Pelvis speed (°/s)	458.3 ± 64.1	428.0 ± 51.3	0.30
Thorax speed (°/s)	712.5 ± 74.1	685.9 ± 62.8	0.43
Lead arm speed (°/s)	1050.5 ± 117.6	947.7 ± 87.3	0.06
Pelvis acceleration (°/s^2^)	2008.2 ± 556.9	1841.3 ± 316.2	0.48
Thorax acceleration (°/s^2^)	3310.9 ± 670.7	3392.7 ± 516.9	0.78
Lead arm acceleration (°/s^2^)	5433.5 ± 1196.7	5135.4 ± 947.2	0.58
X-factor (°)	49.2 ± 9.4	50.9 ± 10.4	0.71
X-factor stretch (°)	7.1 ± 5.5	11.7 ± 7.9	0.14
X-factor stretch rate (°/s)	44.0 ± 36.5	70.7 ± 38.4	0.14
Shoulder stretch (°)	1.7 ± 1.3	1.6 ± 1.5	0.85
*Driver performance*			
Clubhead speed (m/s)	49.1 ± 3.1	41.6 ± 2.3	0.00
Ball speed (m/s)	68.5 ± 4.9	57.1 ± 3.6	0.00
Carry distance (m)	218.5 ± 22.7	179.9 ± 13.6	0.01

Values are mean ± standard deviation and *p*-values are from independent t-tests

Differences before the training intervention between the two training groups IK and IT were also investigated and Table 4 shows that mean age, height, weight and handicap between the two groups were not different at the start of the study. In addition, there was no statistically significant difference between groups before the training study began (*p* > 0.1) for any of the intervention related variables assessed, including rotational and lower body power, swing kinematics and driver performance variables (Table 4).

**Table 4:** Descriptive statistics for the IK and IT group at the start of study

	IK Group (*n* = 10)	IT Group (*n* = 10)	*p*-value
Age (years)	22.0 ± 4.0	22.0 ± 4.0	0.45
Height (cm)	175.0 ± 13.0	178.0 ± 14.0	0.26
Weight (kg)	75.0 ± 22.0	71.0 ± 15.0	0.36
handicap	+0.4 ± 1.0	+0.4 ± 1.7	0.90

Values are mean ± standard deviation and *p*-values are from independent t-tests. *IK* isokinetic training group, *IT* isotonic training group

### Rotational and lower body power

In this study, both dominant and non-dominant side force, velocity, and power were measured in the seated abdominal rotation test. After the nine-week-training period, both the IK and the IT groups increased their dominant side rotational power to a large (ES_w_ = 0.82) and medium (ES_w_ = 0.50) extent respectively (Fig. 1a, Table 5). Between-group ES indicated a small (ES_b_ = 0.32) improvement in favor of the IK group compared to the IT, and MBI indicated that IK had a likely (85%) more beneficial increase in dominant side rotational power compared to the IT (Table 5). Similarly, both training modalities resulted in improvements in dominant side rotational force with a large effect (ES_w_ = 0.96) for the IK group and a medium effect (ES_w_ = 0.77) for the IT group (Table 5). ES_b_ statistics together with MBI demonstrated a near small (ES_b_ = 0.19) but likely (MBI = 80%) more beneficial effect of isokinetic training on dominant side rotational force compared to isotonic strength training (Table 5). For dominant side rotational speed both groups increased with a small (ES_w_ = 0.45, IK) to medium (ES_w_ = 0.5, IT) improvement. However, any difference between groups was considered none to small (Table 5).

**Table 5:** Upper body rotational force, power and velocity and lower body power measurements for pre and post training

	Pre Mean ± SD	Post Mean ± SD	ES_w_	ES_b_	Magnitude of inference		
					Harmful	Trivial	Beneficial
Seated rotation dominant Side							
Force				0.19	16% unlikely	4% very unlikely	80% likely
IK (N)	230.0 ± 53.8	287.7 ± 58.1	0.96				
IT	224.4 ± 68.5	270.7 ± 48.5	0.77				
Power				0.32	14% very unlikely	0% very unlikely	85% likely,
IK (w)	697.5 ± 277.5	942.0 ± 276.4	0.82				
IT	581.3 ± 318.5	731.4 ± 278.7	0.5				
Velocity				−0.11	0% very unlikely	100% most likely	0% very unlikely
IK (m/s)	3.3 ± 0.6	3.6 ± 0.6	0.45				
IT	3.0 ± 0.7	3.4 ± 0.7	0.5				
Seated rotation non-dominant side							
Force (N)				−0.10	65% possibly	6% unlikely	29% possible
IK	239.5 ± 52.0	279.3 ± 37.8	0.71				
IT	214.9 ± 58.4	260.2 ± 58.4	0.82				
Power (W)				0.00	50% possibly	1% very unlikely	49% possibly
IK	725.9 ± 271.9	870.6 ± 247.0	0.5				
IT	586.9 ± 310.0	732.8 ± 289.5	0.5				
Velocity(m/s)				−0.45	0% very unlikely	100% most likely	0% very unlikely
IK	3.28 ± 0.5	3.48 ± 0.7	0.32				
IT	2.89 ± 0.7	3.36 ± 0.7	0.79				
Lower body							
CMJ (cm)				−0.11	45% Possible	45% possibly	10% very unlikely
IK	38.2 ± 8.4	38.0 ± 9.1	−0.03				
IT	43.1 ± 6.4	43.7 ± 6.6	0.08				
LSJ 20 kg (W)				0.02	47% possibly	1% very unlikely	52% possibly
IK	1333.9 ± 209.4	1385.6 ± 227.2	0.22				
IT	1288.4 ± 267.5	1335.5 ± 267.5	0.20				

*SD* standard deviation, *ES* effect size *IK* isokinetic training group, *IT* isotonic training group, *CMJ* counter movement jump, *LSJ* loaded squat jump, *ES*_*w*_ within group ES, *ES*_*b*_ between group ES

Results for force, velocity and power in the non-dominant side rotations were less clear compared to the dominant side. Both the IK and IT groups had medium to large improvements in force and power, but in velocity the IT group increased more compared to the IK group (ES_b_ = −0.45; Table 5). MBI statistics, on the other hand, indicated no clear advantages for either training modality in any of the non-dominant side rotational variables force, velocity or power (Table 5).

For lower body power, both groups responded similarly, where the nine-week-training period had no effect on CMJ (ES_w_ = −0.03 IK and 0.08 IT) and a small effect on 20 kg loaded squat jumps (ES_w_ = 0.22 IK and 0.20 IT) (Table 5).

### Swing kinematics

Results for all measured swing kinematic variables are presented in Table 6. For a number of variables, including shoulder stretch rate (ES_w_ = 0.29 for IK and −0.34 for IT), the groups showed different directions of change (Fig. 1b). After the nine-week training period x-factor stretch (ES_b_ = 0.51), shoulder stretch rate (ES_b_ = 0.63), and arm acceleration (ES_b_ = 0.52) had a likely (≥ 80%) beneficial effect from isokinetic training compared to isotonic strength training (Table 6). Furthermore, lead arm speed had a small increase in both IK (ES_w_ = 0.49) and IT (ES_w_ = 0.24) groups, whereas lead arm acceleration had a small increase only in the IK group (ES_w_ = 0.48). Comparisons of the two groups showed that isokinetic training had a likely more beneficial (> 80%) improvement in lead arm speed and acceleration (ES_b_ = 0.24 and ES_b_ = 0.52, respectively) compared to isotonic strength training (Fig. 1c, Table 6).

**Table 6:** Swing kinematics measurements for pre and post training period

	Pre Mean ± SD	Post Mean ± SD	ES_w_	ES_b_	Magnitude of inference		
					Harmful	Trivial	Beneficial
X-factor (°)				−0.41	73% possibly	12% Unlikely	15% Unlikely,
IK	51.2 ± 10.4	48.7 ± 6.1	−0.26				
IT	48.3 ± 8.8	49.7 ± 14.7	0.15				
X-factor stretch (°)				0.51	4% very unlikely	12% Unlikely	84% Likely
IK	6.9 ± 5.9	8.2 ± 4.2	0.15				
IT	10.5 ± 7.1	8.4 ± 8.6	−0.25				
X-factor stretch rate (°/s)				0.25	25% possibly	4% very Unlikely	71% possibly
IK	48.4 ± 42.6	48.1 ± 31.7	−0.01				
IT	58.3 ± 35.4	48.2 ± 34.3	−0.26				
Shoulder stretch (°)				0.67	2% very unlikely	53% possibly	45% possibly
IK	1.6 ± 1.0	2.0 ± 2.3	0.30				
IT	1.7 ± 1.7	1.2 ± 2.0	−0.37				
Shoulder stretch rate (°/s)				0.63	8% unlikely	4% very unlikely	88% likely
IK	22.4 ± 13.2	26.8 ± 24.2	0.29				
IT	18.6 ± 17.3	13.5 ± 19.1	−0.34				
Lead arm speed (°/s)				0.24	10% unlikely	2% very unlikely	88% likely
IK	1016.2 ± 96.9	1073.8 ± 96.2	0.49				
IT	1012.8 ± 139.5	1042.4 ± 139.6	0.25				
Lead arm acceleration (°/s^2^)				0.52	7% unlikely	0% very unlikely	93% likely
IK	5217.0 ± 943.7	5746.2 ± 658.1	0.48				
IT	5441.3 ± 1278.4	5403.5 ± 1568.3	−0.03				
Thorax speed (°/s)				0.12	29% possibly	4% very unlikely	67% Possibly
IK	697.1 ± 75.4	716.3 ± 53.9	0.28				
IT	709.3 ± 67.3	720.1 ± 77.2	0.16				
Thorax acceleration (°/s^2^)				0.25	29% possibly	0% very unlikely	71% possibly
IK	3228.8 ± 595.0	3302.2 ± 447.3	0.1				
IT	3440.3 ± 636.2	3327.8 ± 873.9	−0.14				
Pelvis speed (°/s)				0.14	32%% possibly	4% very unlikely	64% possibly
IK	441.2 ± 68.2	464.1 ± 61.7	0.38				
IT	454.2 ± 54.4	468.9 ± 61.4	0.24				
Pelvis acceleration (°/s^2^)				−0.07	55% possibly	0% very unlikely	45% possibly
IK	2022.3 ± 269.5	2162.5 ± 439.8	0.29				
IT	1877.3 ± 640.2	2053.0 ± 801.2	0.36				

*SD* standard deviation, *ES* effect size, *K* isokinetic training group, *IT* isotonic training group, *ES*_*w*_ within group ES, *ES*_*b*_ between group ES

### Driver performance variables

After the nine-week training period, CHS showed no improvements in neither the IK (ES_w_ = 0.17) nor the IT (ES_w_ = 0.18) groups (Table 7). However, both IK and IT increased ball speed and carry with isokinetic training showing a small (ES_b_ = 0.21) and possible (65%) more beneficial effect on ball speed when compared to IT and a medium (ES_b_ = 0.59) likely (78%) more beneficial effect on carry distance when compared to IT (Fig. 1d, Table 7).

**Table 7:** Pre and post training period measurements of club head speed, ball speed, and carry distance

	Pre Mean ± SD	Post Mean ± SD	ES_w_	ES_b_	Magnitude of inference		
					Harmful	Trivial	Beneficial
Carry distance (m)				0.31	16% unlikely	6% unlikely	78% likely
IK	197.2 ± 28.6	213.2 ± 34.4	0.59				
IT	212.7 ± 24.8	220.4 ± 23.3	0.28				
Ball speed (m/s)				0.21	4% very unlikely	32% possible	65% possible
IK	63.4 ± 7.6	65.6 ± 6.6	0.32				
IT	65.6 ± 6.7	66.3 ± 6.4	0.11				
Club head speed (m/s)				−0.01	9% Unlikely	84% Likely	7% Unlikely
IK	46.1 ± 4.4	46.9 ± 4.2	0.17				
IT	46.8 ± 5.0	47.7 ± 4.4	0.18				

*SD* standard deviation, *ES* effect size, *IK* isokinetic training group, *IT* isotonic training group, *ES*_*w*_ within group ES, *ES*_*b*_ between group ES

## Discussion

The main findings of this study are that both isokinetic and isotonic strength training over a 9-week period had a moderate to large effect on improving rotational power, force, and velocity in pre-elite golfers. However, with isokinetic training rotational power and force improved more compared to isotonic strength training, whereas speed improved to a similar degree in the two groups. Interesting findings for swing kinematics included the between-group differences in X-factor, X-factor stretch, thorax acceleration, shoulder stretch, and shoulder stretch rate. The larger improvements seen with isokinetic training in rotational power and utilisation of SSC characteristics translated into a higher ball speed, but not into higher CHS, when compared to isotonic strength training.

### Changes in force velocity and power

Many methods exist for increasing muscular force, velocity, and power. We chose to investigate the effects of performing a functional exercise simulating the golf swing using isokinetic training, an area less investigated. Both the IK and IT group improved dominant side rotational force, power, and velocity but IK had a likely (80–85%) larger improvement in force and power compared to IT group. No previous studies have investigated multi-joint isokinetic training in golf performance but results from a recent study using the same isokinetic device found that isokinetic lower body training in different team sport athletes resulted in superior jump and sprint performance when compared to isotonic training [27]. Another study looking at upper-body multi-joint isokinetic training in beginners compared to a non-exercising control group, found significant increases in select upper body exercises in the isokinetic group [29]. Isokinetic training has been proposed to increase the number of motor neurons recruited and produce a more synchronous firing of motor neurons than dynamic training alone [41]. The ability to generate maximal muscular power is considered the most important neuromuscular function in sports performance [42, 43] and the isokinetic training performed by the IK group likely (85%) had a beneficial effect on their dominant side rotational peak power. Previous research comparing effects of resistance training among men and women suggest that, whilst men show greater absolute strength and power, both recreational and elite male and female athletes respond in a similar way to resistance training and power training programs [44–46].

### Changes in kinematics

Improved knowledge of different physical training methods, their importance and impact on the golf swing may allow for a more efficient use of training time. This study implemented only two training exercises into the regular training program of the IK group, one of which was a golf-specific isokinetic rotational movement aimed to mimic the ballistic movements in the golf swing and improve driver performance. Our results presented moderately sized between group differences (ES_b_) for x-factor (0.41), x-factor stretch (0.51), thorax acceleration (0.25), shoulder stretch (0.67), and shoulder stretch rate (0.63) in support of isokinetic training. Further analysis of SSC characteristics showed that IT generally worsened slightly at both the torso and shoulder whilst the IK group showed improvement at the shoulder whilst maintaining SSC parameters in the trunk. Our results for the IT group where small decreases in several swing kinematic variables (ES_w_ 0.25–0.34) were found was rather unexpected since the IT group did improve force, power, and speed in addition to maintaining CHS and BS. The reason for this decrease is difficult to explain but may highlight that a number of different swing techniques are able to maintain CHS and BS. Nevertheless, previous research has shown that increases in strength and power through isotonic training can influence swing kinematics (mechanics) including changes in thorax velocity [21] and x-factor stretch rate [21] and these findings support our results for the IK group, but not for the IT group. Our results demonstrate isokinetic and isotonic strength training programs can modify swing kinematics differently, and IK training appears to be superior for maintaining or improving SSC characteristics among pre-elite golfers with previous experience of isotonic training methods.

### Changes in driver performance

Our results revealed that isokinetic training may have a beneficial effect on carry distance and ball speed, whereas CHS showed no change over the training period for either group. The isotonic training group in the current study saw no increase in either CHS or ball speed, and only a small (ES_w_ = 0.28) increase in carry distance. This is in contrast to strength and plyometric intervention studies on recreational golfers where improvements in CHS, ball speed or driving distance have been found [16–19]. However, it is well documented that eliciting changes amongst an elite population is more difficult [21] and the negligible change in ball speed (1.1%) and CHS (1.9%) found amongst the IT group are similar to findings by Doan et al. [21] who reported a trivial increase of 1.6% in CHS among intercollegiate level golfers after 11 weeks of strength training. Pre-elite golfers with a history of strength training, as in our study, may have already adapted to isotonic training methods and further isotonic training may not elicit further improvements in driver performance.

All participants in this study had an extensive background in isotonic strength training and plyometric training, but little to no prior experience of isokinetic training. The isokinetic group increased in carry distance (7.6%) with no change in CHS (1.7%), which is similar to Fletcher et al. [16] results who also found a greater increase in carry distance (4.3%) compared to CHS (1.5%) from weight and plyometric training among good club golfers with very little prior strength and conditioning experience. We cannot exclude the possibility that the training adaptations seen in our study are in part due to an unaccustomed exercise modality. Nevertheless, we show that isokinetic training elicits additional responses in golfers already well adapted to plyometrics and isotonic training. This is similar to a previous multi-joint isokinetic intervention study in athletes with considerable experience in strength and power training [28]. Carry distance and ball speed are not only dependent on club head velocity, but also centeredness of impact, and clubface orientation [4, 5]. An explanation for the improvement in ball speed and carry distance in the IK group could be that their greater lead arm acceleration resulted in reaching lead arm peak speed earlier in the downswing which may allow for improved centeredness of impact, clubhead path, or clubface orientation at impact. Our results reported increased force development characteristics in both a seated rotation test for power and in the golf swing, possibly suggesting that this increase may transfer into improved centeredness of impact or clubface orientation at impact, greater ball speed, longer carry distance and improved driver performance.

In the current study we included a reference group of highly skilled golfers to account for natural occurring changes in performance during this training period, using an open trial method design, which permitted the participants to self-select experimental group to allow for international competition schedules; both the IK and IT groups had average handicaps better than scratch. There are some uncontrolled variables that may have influenced the training adaptations; for example, the current study did not investigate load and intensity of the normal pre-season training regimens. Both these variables are well known to influence strength and power training adaptations [14, 28]. Furthermore the individualised pre-season training programs or technical swing changes could influence results and should be investigated in future studies. Analyses of the change in size of standard deviation can help describe the change in homogeneity of a group. For instance the IK group showed a decrease in size of standard deviation in pre to post X-factor whilst the IT groups’ standard deviation showed the opposite trend. This suggests that despite individualised training program and different coaches for each participant the IK group became more similar after the training period.

Finally, both IK and IT groups were mixed sexes, and previous cross-sectional studies [24–26] have shown some significant differences in swing kinematics between male and female golfers. In our study we did not find sex-differences in the pelvis, thorax and shoulder kinematic variables before the start of the 9-week training period. Zheng et al. [26] found differences between men and women in maximum velocity of the wrists, right elbow extension, timing of left wrist extension velocity. However, in line with our findings pelvis, trunk, and left arm shoulder abduction angles and velocity were found to be similar between the sexes [26]. Recent research has reported similar improvements in force and rate of velocity development between men and women after isokinetic training [48], which is in line with previous research on isotonic resistance training. However, there is a paucity of research investigating change in swing kinematics among golfers and even fewer studies comparing change in swing kinematics between men and women, an area in need of further investigation.

## Conclusion

Isokinetic training among pre-elite golfers with a history of strength and conditioning training increased rotational power development, SSC characteristics around the shoulder, lead arm peak speed, ball speed, and carry distance more compared to isotonic training. Even though isokinetic training did not increase CHS, it did result in greater carry distance and thus improved driver performance.

## Abbreviations

CHS: Club head speed
CMJ: Countermovement jump
ES_b_: Between-group effect size
ES_w_: Within group effect size
IK: Isokinetic power training group
IT: Isotonic strength training group
MBI: Magnitude-based inference
SSC: Stretch-shortening cycle
